# 2-Amino-6-methyl-1,3-benzothia­zole–octa­nedioic acid (2/1)

**DOI:** 10.1107/S1600536809042652

**Published:** 2009-10-23

**Authors:** Yao-Geng Wang

**Affiliations:** a College of Chemistry and Life Science, Tianjin Key Laboratory of Structure and Performance for Functional Molecule, Tianjin Normal University, Tianjin 300387, People’s Republic of China

## Abstract

Cocrystallization of 2-amino-6-methy-1,3-benzothia­zole with octa­nedioic acid in a mixed methanol–water medium afforded the title 2:1 cocrystal, 2C_8_H_8_N_2_S·C_8_H_14_O_4_. The octa­nedioic acid mol­ecule is located on an inversion centre. In the crystal, inter­molecular N—H⋯O and O—H⋯O hydrogen bonds connect the components into a three-dimensional network.

## Related literature

For mol­ecular self-assembly and its application in crystal engineering, see: Yang *et al.* (2007 ▶); Hunter (1993 ▶); Zhao *et al.* (2007 ▶). For the structures and properties of metal complexes and co-crystals with amino­benzothia­zole and its derivatives, see: Shi *et al.* (2009 ▶); Lynch *et al.* (1999 ▶); Chen *et al.* (2008 ▶); Zhang *et al.* (2009 ▶). For the structure and performance of octa­nedioic acid-based metal complexes and co-crystals, see: Geraghty *et al.* (1999 ▶); McCann *et al.* (1995 ▶); Peral *et al.* (2001 ▶). 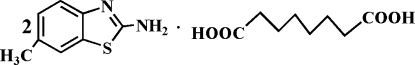


## Experimental

### 

#### Crystal data

2C_8_H_8_N_2_S·C_8_H_14_O_4_
*M* *_r_* = 502.64
Monoclinic, 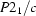

*a* = 12.4372 (12) Å
*b* = 7.9165 (8) Å
*c* = 16.6061 (12) Å
β = 127.992 (5)°
*V* = 1288.6 (2) Å^3^
*Z* = 2
Mo *K*α radiation
μ = 0.24 mm^−1^
*T* = 293 K
0.25 × 0.20 × 0.18 mm

#### Data collection

Bruker APEXII CCD area-detector diffractometer
Absorption correction: multi-scan (*SADABS*; Sheldrick, 1996 ▶) *T*_min_ = 0.942, *T*_max_ = 0.958
6745 measured reflections
2271 independent reflections
1767 reflections with *I* > 2σ(*I*)
*R*_int_ = 0.019

#### Refinement

*R*[*F* ^2^ > 2σ(*F* ^2^)] = 0.033
*wR*(*F* ^2^) = 0.098
*S* = 1.05
2271 reflections
156 parameters
H-atom parameters constrained
Δρ_max_ = 0.16 e Å^−3^
Δρ_min_ = −0.20 e Å^−3^

### 

Data collection: *APEX2* (Bruker, 2003 ▶); cell refinement: *SAINT* (Bruker, 2001 ▶); data reduction: *SAINT*; program(s) used to solve structure: *SHELXS97* (Sheldrick, 2008 ▶); program(s) used to refine structure: *SHELXL97* (Sheldrick, 2008 ▶); molecular graphics: *SHELXTL* (Sheldrick, 2008 ▶) and *DIAMOND* (Brandenburg & Berndt, 1999 ▶); software used to prepare material for publication: *SHELXL97*.

## Supplementary Material

Crystal structure: contains datablocks I, global. DOI: 10.1107/S1600536809042652/bt5102sup1.cif

Structure factors: contains datablocks I. DOI: 10.1107/S1600536809042652/bt5102Isup2.hkl

Additional supplementary materials:  crystallographic information; 3D view; checkCIF report

## Figures and Tables

**Table 1:** Hydrogen-bond geometry (Å, °)

*D*—H⋯*A*	*D*—H	H⋯*A*	*D*⋯*A*	*D*—H⋯*A*
O2—H2⋯N1	0.82	1.79	2.5973 (19)	169
N2—H2*B*⋯O1^i^	0.86	2.10	2.922 (2)	159
N2—H2*A*⋯O1	0.86	2.19	3.009 (2)	160

Symmetry code: (i) 
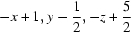
.

**Fig. 1.**
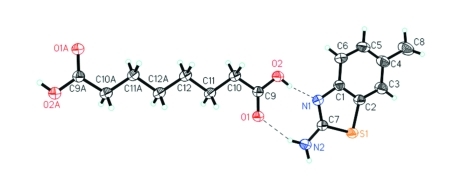
The molecular structure of the title compound. Displacement ellipsoids are drawn at the 30% probability level. The dashed lines indicate intermolecular hydrogen bonds. Symmetry code: (A) 1 – x, 2 – y, 2 – z.

## supplementary crystallographic information

### Comment

Nowadays, molecular self-assembly driven by popular coordination bonds and weak intermolecular non-covalent interactions (hydrogen-bonding, π···π stack, electrostatic interactions and so on), has been attracting more and more interest in biochemistry, life science and new material fields (Hunter, 1993; Yang *et al.*, 2007; Zhao *et al.*, 2007). In this regard, aminobenzothiazole and its varios derivatives have been becoming one of the excellent building blocks with multiple hydrogen-bonding and metal ion binding sites and have been extensively applied in new materials, biochemistry and agriculture chemistry, due to the lower toxicity, high biological activity and excellent chemical reactivity (Shi *et al.*, 2009; Lynch *et al.*, 1999; Chen *et al.*, 2008; Zhang *et al.*, 2009).On the other hand, the long octanedioic acid with variable deprotonated form and flexible aliphatic chain has also exhibited novel functions in the fields of metal complexes and molecular co-crystals (McCann *et al.* 1995; Peral *et al.* 2001; Geraghty *et al.* 1999).

Herein, as a continuation of molecular assembly behavior in the solid state, the rigid 2-amino-6-methy-1,3-benzothiazole and flexible octanedioic acid were selected as building blocks to cocrystallize. Consequently, an intermolecular hydrogen bonded adduct, (I), was obtained in the mixed methanol-water medium, exhibiting three-dimensional   network by intermolecular hydrogen-bonding interactions.

As shown in Fig. 1, the asymmetric unit of (I) contains one neutral 2-amino-6-methy-1,3-benzothiazole molecule with no crystallographically imposed symmetry and half a octanedioic acid located on a centre of inversion. Obviously, no proton transfer was observed for the neutral cocrystal, which is different from the 2-aminobenzothiazolium 2,4-dicarboxybenzoate monohydrate (Zhang *et al., *2009). The exocyclic amino group of 2-amino-6-methy-1,3-benzothiazole is roughly coplanar with the benzothiazole ring. Similarily, the carboxylic residues of octanedioic acid are also co-planar with their long aliphatic chain. In the packing structure of I, two pairs of the intermolecuar O2—H2 ···N1 and N2—H2A ···O1 hydrogen-bonding interactions (Table 1) connect the two 2-amino-6-methy-1,3-benzothiazole molecules and one octanedioic acid into a trimer. Furthermore, the adjacent trimers are hydrogen-bonded together by N2—H2B···O1 to generate a three dimensional network.

### Experimental

2-Amino-6-methylbenzothiazole (16.4 mg, 0.1 mmol) and octanedioic acid (17.4 mg, 0.1 mmol) were dissolved in a mixed methanol-water solution (1:1, 10 ml). The resulting mixture was stirring for one hour and filtered. The colorless filtrate was left to stand at room temperature. The colorless block-shaped crystals suitable for *x*-ray diffraction were isolated by slow evaporation of the solvent in one week (yield: 30.0% based on 2-amino-6-methylbenzothiazole). Analysis calculated for C_48_H_60_N_8_O_8_S_4_: C 57.35, H 6.02, N 11.15%; found: C 57.55, H 6.00, N 11.48%.

### Refinement

H-atoms were located in difference maps, but were subsequently placed in calculated positions and treated as riding, with C–H = 0.93 (aromatic) or 0.96 (methyl and methylene)Å, O – H = 0.82 Å, and N – H = 0.86 Å. All H atoms were allocated displacement parameters related to those of their parent atoms [*U*_iso_(H)] = 1.2 *U*_eq_ (C, N, O) or *U*_iso_(H)] = 1.5 *U*_eq_ (C_methyl_)].

### Crystal data

**Table:**

2C_8_H_8_N_2_S·C_8_H_14_O_4_	*F*(000) = 532
*M**_r_* = 502.64	*D*_x_ = 1.295 Mg m^−^^3^
Monoclinic, *P*2_1_/*c*	Mo *K*α radiation, λ = 0.71073 Å
*a* = 12.4372 (12) Å	Cell parameters from 2130 reflections
*b* = 7.9165 (8) Å	θ = 2.5–24.4°
*c* = 16.6061 (12) Å	µ = 0.24 mm^−^^1^
β = 127.992 (5)°	*T* = 293 K
*V* = 1288.6 (2) Å^3^	Block, colourless
*Z* = 2	0.25 × 0.20 × 0.18 mm

### Data collection

**Table:**

Bruker APEXII CCD area-detector diffractometer	2271 independent reflections
Radiation source: fine-focus sealed tube	1767 reflections with *I* > 2σ(*I*)
graphite	*R*_int_ = 0.019
φ and ω scans	θ_max_ = 25.0°, θ_min_ = 2.1°
Absorption correction: multi-scan (*SADABS*; Sheldrick, 1996)	*h* = −14→13
*T*_min_ = 0.942, *T*_max_ = 0.958	*k* = −7→9
6745 measured reflections	*l* = −19→19

### Refinement

**Table:**

Refinement on *F*^2^	Primary atom site location: structure-invariant direct methods
Least-squares matrix: full	Secondary atom site location: difference Fourier map
*R*[*F*^2^ > 2σ(*F*^2^)] = 0.033	Hydrogen site location: inferred from neighbouring sites
*wR*(*F*^2^) = 0.098	H-atom parameters constrained
*S* = 1.05	*w* = 1/[σ^2^(*F*_o_^2^) + (0.0501*P*)^2^ + 0.215*P*] where *P* = (*F*_o_^2^ + 2*F*_c_^2^)/3
2271 reflections	(Δ/σ)_max_ = 0.001
156 parameters	Δρ_max_ = 0.16 e Å^−^^3^
0 restraints	Δρ_min_ = −0.20 e Å^−^^3^

### Special details

**Table:**

Geometry. All e.s.d.'s (except the e.s.d. in the dihedral angle between two l.s. planes) are estimated using the full covariance matrix. The cell e.s.d.'s are taken into account individually in the estimation of e.s.d.'s in distances, angles and torsion angles; correlations between e.s.d.'s in cell parameters are only used when they are defined by crystal symmetry. An approximate (isotropic) treatment of cell e.s.d.'s is used for estimating e.s.d.'s involving l.s. planes.
Refinement. Refinement of *F*^2^ against ALL reflections. The weighted *R*-factor *wR* and goodness of fit *S* are based on *F*^2^, conventional *R*-factors *R* are based on *F*, with *F* set to zero for negative *F*^2^. The threshold expression of *F*^2^ > σ(*F*^2^) is used only for calculating *R*-factors(gt) *etc*. and is not relevant to the choice of reflections for refinement. *R*-factors based on *F*^2^ are statistically about twice as large as those based on *F*, and *R*- factors based on ALL data will be even larger.

### Fractional atomic coordinates and isotropic or equivalent isotropic displacement parameters (Å^2^)

**Table:**

	*x*	*y*	*z*	*U*_iso_*/*U*_eq_	
S1	0.27303 (5)	−0.11551 (6)	1.15536 (3)	0.06084 (19)	
O1	0.41522 (14)	0.48915 (16)	1.10448 (9)	0.0707 (4)	
O2	0.24755 (15)	0.39416 (17)	0.95120 (9)	0.0743 (4)	
H2	0.2495	0.3168	0.9849	0.111*	
N1	0.23024 (15)	0.12817 (17)	1.03421 (11)	0.0561 (4)	
N2	0.40617 (17)	0.1802 (2)	1.20575 (12)	0.0760 (5)	
H2A	0.4222	0.2778	1.1924	0.091*	
H2B	0.4541	0.1450	1.2679	0.091*	
C1	0.13613 (17)	0.0017 (2)	0.97271 (13)	0.0517 (4)	
C2	0.14393 (17)	−0.1411 (2)	1.02435 (13)	0.0534 (4)	
C3	0.0586 (2)	−0.2780 (3)	0.97316 (14)	0.0700 (6)	
H3	0.0656	−0.3730	1.0090	0.084*	
C4	−0.0376 (2)	−0.2717 (3)	0.86769 (15)	0.0711 (6)	
C5	−0.0446 (2)	−0.1297 (3)	0.81731 (15)	0.0716 (6)	
H5	−0.1090	−0.1260	0.7465	0.086*	
C6	0.03990 (19)	0.0074 (3)	0.86719 (13)	0.0656 (5)	
H6	0.0325	0.1020	0.8308	0.079*	
C7	0.30726 (18)	0.0837 (2)	1.13027 (13)	0.0541 (4)	
C8	−0.1328 (3)	−0.4193 (4)	0.81008 (19)	0.1066 (9)	
H8A	−0.1898	−0.3967	0.7381	0.160*	
H8B	−0.1888	−0.4354	0.8311	0.160*	
H8C	−0.0803	−0.5196	0.8246	0.160*	
C9	0.33968 (18)	0.5053 (2)	1.01209 (13)	0.0537 (4)	
C10	0.34445 (19)	0.6517 (2)	0.95779 (13)	0.0570 (5)	
H10A	0.2554	0.7052	0.9163	0.068*	
H10B	0.3616	0.6092	0.9118	0.068*	
C11	0.45049 (18)	0.7847 (2)	1.02578 (12)	0.0541 (4)	
H11A	0.5402	0.7330	1.0659	0.065*	
H11B	0.4351	0.8265	1.0728	0.065*	
C12	0.44826 (18)	0.9325 (2)	0.96670 (13)	0.0564 (4)	
H12A	0.4649	0.8906	0.9204	0.068*	
H12B	0.3580	0.9825	0.9257	0.068*	

### Atomic displacement parameters (Å^2^)

**Table:**

	*U*^11^	*U*^22^	*U*^33^	*U*^12^	*U*^13^	*U*^23^
S1	0.0674 (3)	0.0618 (3)	0.0453 (3)	−0.0065 (2)	0.0307 (2)	0.0036 (2)
O1	0.0878 (9)	0.0557 (8)	0.0427 (7)	−0.0119 (7)	0.0270 (7)	0.0017 (6)
O2	0.0888 (10)	0.0590 (9)	0.0466 (7)	−0.0179 (7)	0.0274 (7)	0.0011 (6)
N1	0.0597 (9)	0.0503 (9)	0.0469 (8)	0.0005 (7)	0.0272 (7)	0.0031 (7)
N2	0.0856 (12)	0.0609 (10)	0.0485 (9)	−0.0145 (9)	0.0246 (9)	−0.0005 (8)
C1	0.0489 (9)	0.0540 (10)	0.0480 (9)	0.0032 (8)	0.0276 (8)	0.0002 (8)
C2	0.0515 (10)	0.0613 (11)	0.0464 (9)	−0.0034 (8)	0.0296 (8)	0.0004 (8)
C3	0.0740 (13)	0.0726 (14)	0.0621 (12)	−0.0200 (11)	0.0413 (11)	−0.0048 (10)
C4	0.0610 (12)	0.0830 (15)	0.0565 (11)	−0.0164 (11)	0.0296 (10)	−0.0111 (11)
C5	0.0592 (12)	0.0876 (16)	0.0461 (10)	−0.0005 (11)	0.0215 (9)	−0.0039 (11)
C6	0.0621 (11)	0.0698 (13)	0.0470 (10)	0.0057 (10)	0.0245 (9)	0.0068 (9)
C7	0.0588 (10)	0.0519 (10)	0.0460 (9)	0.0016 (8)	0.0294 (9)	0.0009 (8)
C8	0.0974 (18)	0.117 (2)	0.0769 (16)	−0.0487 (16)	0.0392 (14)	−0.0237 (15)
C9	0.0618 (11)	0.0464 (10)	0.0449 (10)	0.0025 (8)	0.0288 (9)	0.0002 (8)
C10	0.0641 (11)	0.0534 (10)	0.0465 (9)	0.0020 (9)	0.0304 (9)	0.0046 (8)
C11	0.0611 (11)	0.0489 (10)	0.0479 (9)	0.0039 (8)	0.0313 (9)	0.0055 (8)
C12	0.0618 (11)	0.0547 (10)	0.0475 (9)	0.0039 (8)	0.0310 (9)	0.0085 (8)

### Geometric parameters (Å, °)

**Table:**

S1—C2	1.7469 (18)	C5—C6	1.377 (3)
S1—C7	1.7491 (19)	C5—H5	0.9300
O1—C9	1.2159 (19)	C6—H6	0.9300
O2—C9	1.297 (2)	C8—H8A	0.9600
O2—H2	0.8200	C8—H8B	0.9600
N1—C7	1.306 (2)	C8—H8C	0.9600
N1—C1	1.394 (2)	C9—C10	1.492 (2)
N2—C7	1.331 (2)	C10—C11	1.513 (2)
N2—H2A	0.8599	C10—H10A	0.9700
N2—H2B	0.8601	C10—H10B	0.9700
C1—C2	1.386 (2)	C11—C12	1.516 (2)
C1—C6	1.387 (2)	C11—H11A	0.9700
C2—C3	1.383 (3)	C11—H11B	0.9700
C3—C4	1.386 (3)	C12—C12^i^	1.507 (4)
C3—H3	0.9300	C12—H12A	0.9700
C4—C5	1.371 (3)	C12—H12B	0.9700
C4—C8	1.513 (3)		
			
C2—S1—C7	88.84 (8)	C4—C8—H8A	109.5
C9—O2—H2	109.5	C4—C8—H8B	109.5
C7—N1—C1	110.78 (15)	H8A—C8—H8B	109.5
C7—N2—H2A	120.0	C4—C8—H8C	109.5
C7—N2—H2B	120.0	H8A—C8—H8C	109.5
H2A—N2—H2B	120.0	H8B—C8—H8C	109.5
C2—C1—C6	119.08 (17)	O1—C9—O2	122.54 (16)
C2—C1—N1	115.16 (15)	O1—C9—C10	123.85 (16)
C6—C1—N1	125.76 (17)	O2—C9—C10	113.60 (15)
C3—C2—C1	121.59 (17)	C9—C10—C11	115.48 (14)
C3—C2—S1	128.73 (15)	C9—C10—H10A	108.4
C1—C2—S1	109.67 (13)	C11—C10—H10A	108.4
C2—C3—C4	119.16 (19)	C9—C10—H10B	108.4
C2—C3—H3	120.4	C11—C10—H10B	108.4
C4—C3—H3	120.4	H10A—C10—H10B	107.5
C5—C4—C3	118.79 (19)	C10—C11—C12	113.19 (14)
C5—C4—C8	121.09 (19)	C10—C11—H11A	108.9
C3—C4—C8	120.1 (2)	C12—C11—H11A	108.9
C4—C5—C6	122.73 (18)	C10—C11—H11B	108.9
C4—C5—H5	118.6	C12—C11—H11B	108.9
C6—C5—H5	118.6	H11A—C11—H11B	107.8
C5—C6—C1	118.65 (19)	C12^i^—C12—C11	113.92 (17)
C5—C6—H6	120.7	C12^i^—C12—H12A	108.8
C1—C6—H6	120.7	C11—C12—H12A	108.8
N1—C7—N2	123.60 (17)	C12^i^—C12—H12B	108.8
N1—C7—S1	115.54 (13)	C11—C12—H12B	108.8
N2—C7—S1	120.86 (14)	H12A—C12—H12B	107.7
			
C7—N1—C1—C2	−0.4 (2)	C8—C4—C5—C6	179.7 (2)
C7—N1—C1—C6	−179.52 (17)	C4—C5—C6—C1	0.1 (3)
C6—C1—C2—C3	0.3 (3)	C2—C1—C6—C5	−0.2 (3)
N1—C1—C2—C3	−178.90 (17)	N1—C1—C6—C5	178.93 (18)
C6—C1—C2—S1	179.83 (14)	C1—N1—C7—N2	179.78 (17)
N1—C1—C2—S1	0.64 (19)	C1—N1—C7—S1	0.0 (2)
C7—S1—C2—C3	178.97 (19)	C2—S1—C7—N1	0.34 (15)
C7—S1—C2—C1	−0.53 (13)	C2—S1—C7—N2	−179.49 (17)
C1—C2—C3—C4	−0.4 (3)	O1—C9—C10—C11	−0.7 (3)
S1—C2—C3—C4	−179.81 (16)	O2—C9—C10—C11	−179.99 (16)
C2—C3—C4—C5	0.3 (3)	C9—C10—C11—C12	−178.51 (16)
C2—C3—C4—C8	−179.5 (2)	C10—C11—C12—C12^i^	179.07 (19)
C3—C4—C5—C6	−0.2 (3)		

Symmetry codes: (i) −*x*+1, −*y*+2, −*z*+2.

### Hydrogen-bond geometry (Å, °)

**Table:**

*D*—H···*A*	*D*—H	H···*A*	*D*···*A*	*D*—H···*A*
O2—H2···N1	0.82	1.79	2.5973 (19)	169
N2—H2B···O1^ii^	0.86	2.10	2.922 (2)	159
N2—H2A···O1	0.86	2.19	3.009 (2)	160

Symmetry codes: (ii) −*x*+1, *y*−1/2, −*z*+5/2.
